# A rare case of littoral cell angioma with paroxysmal nocturnal hemoglobinuria

**DOI:** 10.1097/MD.0000000000046255

**Published:** 2026-05-12

**Authors:** Zhenpeng Li, Sensen Zhang, Jun Wu, Zhonghai Du, Shuxian Niu

**Affiliations:** a First Clinical Medical College of Shandong University of Traditional Chinese Medicine, Jinan City, Shandong Province, China; b Weifang Municipal Hospital of Traditional Chinese Medicine, Weifang, Shandong Province, China.

**Keywords:** case report, laparoscopic splenectomy, littoral cell angioma, paroxysmal nocturnal hemoglobinuria

## Abstract

**Rationale:**

We report a rare case of littoral cell angioma (LCA) in a patient with paroxysmal nocturnal hemoglobinuria (PNH). To our knowledge, this is the first report of these 2 rare conditions occurring together, which may help clarify their relationship and its importance in clinical practice.

**Patient concerns:**

A 38-year-old man had recurrent anemia and dark brown urine, accompanied by abdominal pain that worsened over time, which required several hospital visits and long-term follow-up.

**Diagnoses:**

Follow-up scans showed progressive splenomegaly with multiple nodules. In combination with histopathology and immunohistochemistry results (CD31, CD34, and ERG positive; Ki-67 around 5%), these findings confirmed a final diagnosis of LCA in a patient with previously diagnosed PNH.

**Interventions:**

The patient received treatment with oral prednisone, sodium bicarbonate, and vitamin E for PNH. As the splenic lesions enlarged and blood counts worsened, he subsequently underwent laparoscopic splenectomy in February 2025.

**Outcomes:**

After surgery, both LCA and anemia concurrently resolved, with normalization of hematologic parameters and resolution of abdominal symptoms.

**Lessons:**

This case suggests a potential association between LCA and PNH. Splenectomy provided dual benefits by treating the splenic tumor and improving hemolysis. Given the possible risk of malignant transformation, long-term follow-up is recommended.

## 1. Introduction

Littoral cell angioma (LCA), first identified and named by Falk in 1991,^[1]^ is an uncommon benign tumor arising from the littoral cells of the splenic red pulp, displaying both endothelial and histiocytic differentiation. Although its pathophysiology is not fully understood, growing data indicates possible links to immunological dysregulation, malignant transformation, and chronic inflammation.^[2,3]^ Paroxysmal nocturnal hemoglobinuria (PNH) is a rare acquired clonal condition of hematopoietic stem cells, marked by complement-mediated intravascular hemolysis, a tendency for thrombosis, and bone marrow failure. The primary mechanism entails a mutation in the PIG-A gene, leading to a lack of complement regulating proteins on the cell membrane (such as CD55/CD59), which ultimately triggers abnormal complement activation and erythrocyte destruction.^[4]^ Despite PNH and LCA being separate disease disorders, both exhibit close associations with immune system dysfunction. Recent studies have identified possible correlations between LCA and certain immune-related illnesses, such as hemolytic anemia and idiopathic thrombocytopenia, as well as malignancies; however, no documented instances of concurrent PNH and LCA have been recorded.^[5]^ This article describes a distinctive case of a 38-year-old individual exhibiting simultaneous PNH and LCA. The advancement of LCA throughout the clinical course shown a substantial connection with the aggravation of hemolytic anemia in PNH. We propose that dysregulation of the autoimmune system and localized chronic inflammation in the spleen may contribute to the onset and progression of LCA, based on a thorough study of clinical presentations, imaging developments, and pathological processes. The lack of CD59 expression in splenic tissue necessitates additional investigation to corroborate this theory. Moreover, the hypersplenism and modified immunological microenvironment caused by LCA may aggravate PNH-related hemolysis, creating a harmful feedback loop. This case offers clinical evidence for the interaction mechanism between PNH and LCA, while also confirming the dual therapeutic effectiveness of splenectomy in improving the comorbid condition of both diseases, highlighting the necessity for long-term monitoring of malignant transformation and complications related to LCA.

## 2. Case description

**Figure 1.**
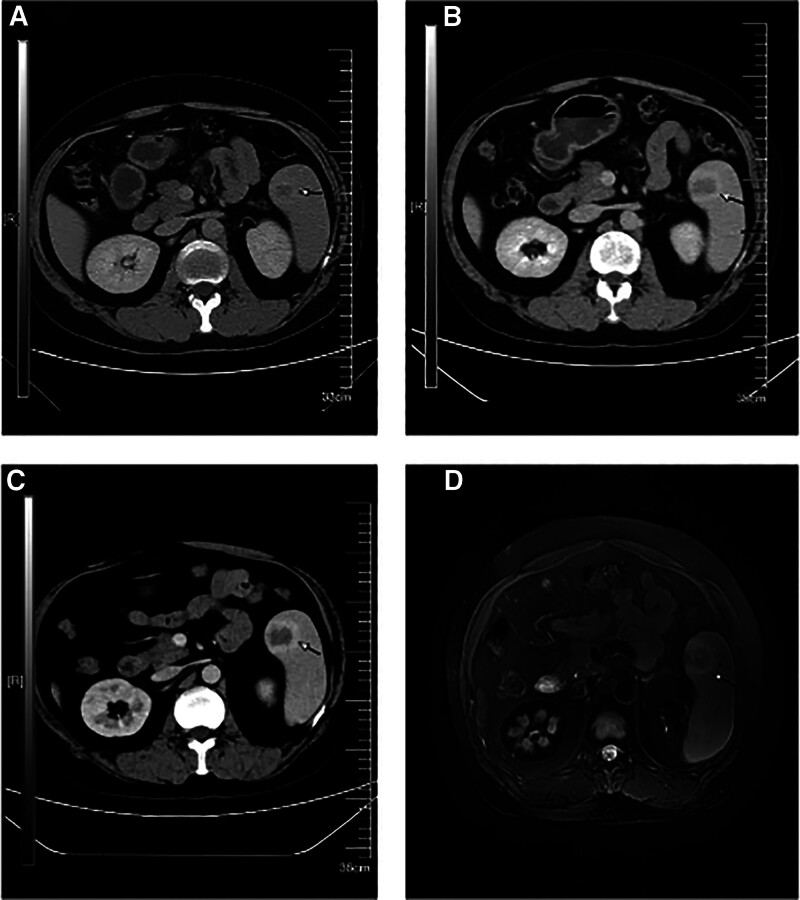
Enhanced CT reveals a localized lesion in the spleen, indicated by the arrow at the lesion site. (A) 2020 Connecticut (B) 2024 Connecticut (C) 2025 CT (D) MRI 2025. CT = computed tomography.

**Figure 2.**
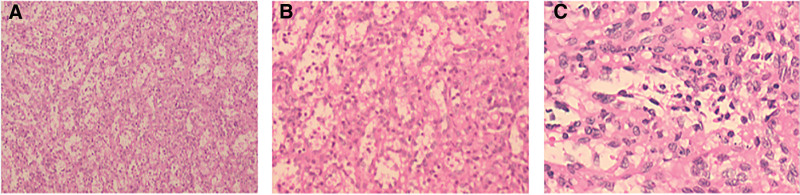
Pathological examination of surgically resected tissue. Hematoxylin-eosin staining revealed perivascular lymphocytic infiltration in the white pulp, characterized by a homogeneous distribution of white pulp, dilated sinusoids in the localized area, and hyperplasia of endothelial cells. (A) Magnification is 100, (B) magnification is 200, (C) magnification is 400.

**Figure 3.**
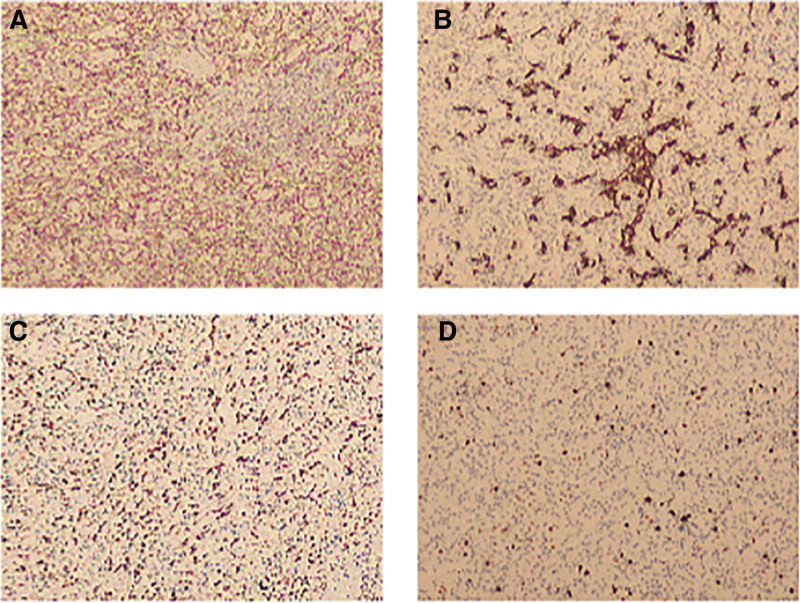
Immunohistochemical examination of tumor tissue specimens post-total splenectomy in 2025. (A) CD31 (+), (B) CD34 (+), (C) ERG (+), (D) Ki-67 5%.

**Figure 4.**
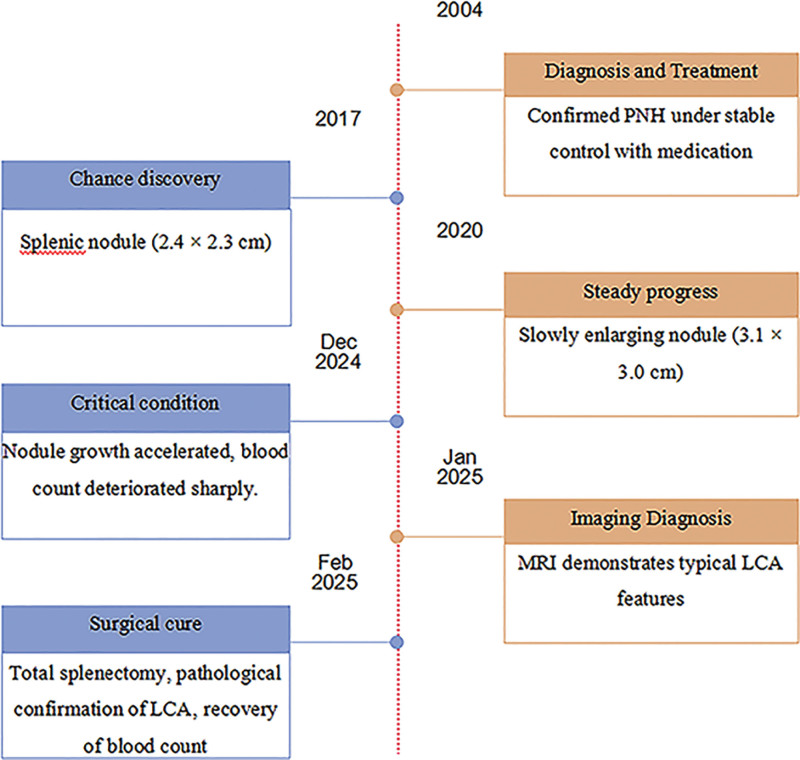
Timeline of critical periods in disease progression.

A 38-year-old male patient went to the hospital in 2004 with symptoms of anemia and black urine. Laboratory studies indicated significant hematological parameters: erythrocyte count (1.89 × 10^12^/L)(4.0–5.5 × 10^12^/L), hemoglobin concentration (41 g/L)(120–160 g/L), and leukocyte count (2.38 × 10^9^/L)(4.0–10.0 × 10^9^/L). Flow cytometric examination revealed a significant prevalence of PNH clones in erythrocytes (type II + type III) at 82.61%, with type III clones with total CD59 deficiency constituting 82.13%. The percentage of PNH clones in granulocytes (type II + type III) was 94.71%. A conclusive diagnosis of PNH was confirmed, and the patient was sustained on a sporadic regimen of oral prednisone, sodium bicarbonate, and vitamin E supplementation, leading to the alleviation of anemia and the elimination of dark urine. In 2017, the patient was readmitted for longitudinal follow-up, presenting with stomach pain. Contrast-enhanced CT imaging demonstrated splenomegaly with a subcapsular nodule measuring 2.4 × 2.3 cm, with radiological features indicative of a hemangioma (sinusoidal cell type not eliminated). The follow-up abdomen CT in 2020 revealed an increase in the nodule size to 3.1 × 3.0 cm (Fig. 1A). By December 2024, upper abdomen CT imaging indicated an increase in the lesion size to 3.6 × 3.2 cm (Fig. 1B), concurrent with the onset of refractory cytopenia (hemoglobin decreased from 89 g/L to 54 g/L, and platelet count fell from 67 × 10⁹/L to 26 × 10⁹/L [100–300 × 10⁹/L]). In January 2025, contrast-enhanced MRI demonstrated numerous splenic nodules, the largest measuring 3.8 × 3.0 cm, exhibiting hypointensity on T1-weighted imaging and hyperintensity on T2-weighted imaging with centripetal enhancement (Fig. 1D). A follow-up abdomen CT in February 2025 revealed additional advancement of the prominent nodule to 4.0 × 3.6 cm (Fig. 1C), consistent with the radiological characteristics of sinusoidal cell hemangioma (LCA). As a result, laparoscopic complete splenectomy was conducted on February 24, 2025. Histopathological analysis indicated sinusoidal cell proliferation in the spleen, with immunohistochemistry staining showing positive for CD31, CD34, and ERG, alongside a Ki-67 proliferation index of 5%, thereby confirming the diagnosis of LCA (Figs. 2 and 3). The postoperative course was uneventful, with no need for blood transfusions, and hematological parameters progressively returned to normal (Fig. 4).

## 3. Discussion

LCA is an uncommon primary benign tumor of the spleen, derived from the sinusoidal cells of the splenic red pulp. The pathophysiology and biological behavior of this organism are not fully understood, and study literature is scarce. Nonetheless, recent research indicates possible correlations with immune system dysregulation, malignant tumors, and chronic inflammatory conditions.^[2,3]^ Troung et al have proposed that LCA may signify a reactive pathogenic condition. The spleen, as a specialized immune filtration organ, filters circulating tumor cells and immune complexes, potentially eliciting a vascular proliferative response.^[6]^ The median age of onset is 45 years, with no notable gender preference. The majority of individuals are asymptomatic, although a few may exhibit indications of hypersplenism, such as splenomegaly, thrombocytopenia, or anemia, sometimes accompanied by stomach pain or splenic rupture.^[5,7]^ The most notable imaging feature is splenomegaly accompanied by several gradually intensifying nodules.^[8]^ LCA is often discovered inadvertently during the assessment of abdominal symptoms or through imaging techniques.^[9,10]^ Pathologically, LCA is marked by diffuse numerous nodules (with indistinct borders) or solitary nodules, wherein tumor cells have dual differentiation traits of both endothelial and histiocytic lineages.^[11–13]^ Unlike other hemangiomas, LCA may have positive staining for transferrin receptor, as well as positive immunohistochemistry markers for endothelial cells and histiocytes, including factor VIII (+), CD31 (+), CD163 (+), CD68 (+), and CD21 (+).^[14]^ The enduring negativity of CD8 differentiates LCA from normal sinusoidal cells.^[15]^ For splenic tumors without malignant potential, it is advisable to conduct routine follow-up every 3 to 6 months. Biopsy is necessary for lesions of indeterminate etiology or suspected cancer. Upon confirmation of the LCA diagnosis, ongoing observation is important. In instances exhibiting malignant transformation potential, notable clinical signs, or progressive tumor growth, splenectomy is the preferred treatment option.^[12]^

PNH was first delineated by J. Enneking in 1928. The primary pathophysiology of this condition arises from mutations in the PIG-A gene in hematopoietic stem cells, leading to a lack of complement regulating proteins on the cellular membrane, including CD55 and CD59. This molecular anomaly induces aberrant activation of the complement system and the subsequent development of the membrane attack complex, ultimately resulting in erythrocyte destruction.^[16]^ The clinical symptoms of PNH mostly include hemolytic anemia, thrombotic events, and smooth muscle dystonia, with certain instances possibly advancing to bone marrow failure.^[17]^ Therapeutic approaches for PNH are dictated by the clinical categorization of the condition. The primary treatment for typical PNH consists of complement inhibitors, such as eculizumab, ravulizumab, and factor B inhibitors.^[4,17,18]^

The simultaneous presence of PNH and lymphangioleiomyomatosis (LCA) constitutes an exceedingly rare clinical condition. This case report details a patient with PNH and concomitant Lymphocytic Choriomeningitis (LCA) who experienced refractory anemia. Throughout the maintenance therapy for PNH, the patient’s hematological values consistently stayed within normal limits. A steady worsening of anemia was noted, resulting in the diagnosis of LCA via imaging techniques. Considering that LCA might itself provoke anemia, we propose that the aggravation of anemia correlates with the development of LCA. Current data indicates that LCA may worsen anemia by increasing splenic plasma pools and causing platelet entrapment within the proliferative network, without a corresponding rise in circulating blood volume, therefore exacerbating anemia.^[5]^ We performed an examination of the relationship between LCA and PNH regarding the evolution of anemia. The progression of LCA leads to splenomegaly, which intensifies the breakdown of erythrocytes by PNH, hence exacerbating anemia. To yet, no conclusive research has clarified the link and interaction mechanisms between PNH and LCA. LCA has been linked to immune-mediated disorders or systemic diseases that induce immunological dysregulation, such as idiopathic thrombocytopenia, chronic hepatitis C, hemolytic anemia, autoimmune thrombocytopenia (Evans syndrome), ulcerative colitis, and ankylosing spondylitis.^[11]^ CD59, found on the endothelial cells of organs like the spleen,^[19]^ is essential for safeguarding splenic and other autologous tissues.^[20]^ We hypothesize that the absence of CD59 may increase the susceptibility of splenic sinus endothelial cells to complement-mediated assault, thereby initiating local vascular reactions and facilitating the establishment of LCA. In addition, previous studies have reported that inflammation greatly promotes tumor initiation and progression.^[21]^ In PNH, abnormal complement activation can effectively induce neutrophil activation, which in turn drives systemic inflammation.^[22]^ At the same time, reports have indicated that the pathogenesis of LCA is associated with chronic inflammation.^[2,3]^ These findings add further evidence that the development of LCA is closely related to PNH. Given the patient’s worsening anemia and the documented safety and postoperative advantages of laparoscopic splenectomy, we advocate for splenectomy as the primary treatment approach for LCA patients exhibiting abdominal discomfort, ambiguous pathology, or advancing tumor growth.^[12]^ The patient has not previously received complement inhibitor therapy for PNH due to economic restrictions and restricted pharmacological availability. The decision to do splenectomy was chiefly motivated by the advancement of LCA and concomitant splenomegaly. After thorough assessment, the patient successfully underwent laparoscopic splenectomy without any postoperative problems. Postoperative results indicated enhancement in both LCA and hemolytic anemia, however the PNH clone remained. A systematic decrement in corticosteroid dosage was executed, warranting continuous long-term monitoring. This example exemplifies the dual therapeutic function of laparoscopic splenectomy in addressing this intricate clinical scenario.

The literature indicates an increased risk of malignant neoplasms linked to lymphangioleiomyomatosis (LCA).^[3,5,7]^ The range of commonly encountered malignancies includes epithelial, mesenchymal, and hematological neoplasms, such as lymphoma, colorectal adenocarcinoma, pancreatic carcinoma, renal cell carcinoma, malignant melanoma, gastric leiomyosarcoma, and non-small cell lung cancer. A retrospective case study by Peckova et al demonstrated a notable correlation between LCA and several visceral cancers, with an incidence rate of 60%.^[2]^ In this case, a review of the patient’s medical history revealed a 3.2 × 2.8 cm space-occupying lesion in the right pulmonary hilum, identified during a comprehensive abdominal CT scan conducted in December 2017 due to abdominal pain. The pathological examination after biopsy verified the lack of malignant cells, and the subsequent follow-up indicated lesion stability. Although the pulmonary lesion in this case was benign and showed no clear link to LCA, the recognized connection between LCA and malignant tumors requires increased awareness for possible malignancies in other organ systems. Therefore, the establishment of routine follow-up measures and ongoing monitoring is essential after splenectomy.^[9]^ Several indicators have been studied and applied to evaluate the occurrence, progression, and prognosis of cance, including the Royal Marsden Hospital score (which combines albumin, lactate dehydrogenase [LDH], and metastatic burden) and the neutrophil-to-eosinophil ratio (NER).^[23,24]^ They have been shown to have some reference value in determining the prognosis of cancer, especially for solid tumors like gastrointestinal tract tumors. In this patient, abnormalities in albumin levels and NER were consistently observed at different stages of disease monitoring, providing retrospective support for their association with LCA. These findings suggest that abnormal Royal Marsden Hospital scores and NER may serve as useful markers for tracking the development, progression, and prognosis of this disease.

## 4. Conclusion

This article reports the inaugural reported instance of PNH occurring along with lymphangioleiomyomatosis (LCA). The CD59 deficit linked to PNH may undermine the splenic immunological barrier, thereby promoting the onset of LCA. Simultaneously, hypersplenism resulting by LCA intensifies the hemolytic process in PNH, creating a pathophysiological vicious cycle. In this clinical case, laparoscopic splenectomy significantly mitigated hypersplenism and corrected the dysfunctional immunological milieu, concurrently improving both LCA-associated anemia and PNH-induced hemolysis. The treatment efficacy of this method in individuals with significant PNH-LCA comorbidity requires additional examination. Despite the generally benign nature of LCA, the possible danger of concomitant malignancies requires diligent monitoring (the lung mass in this instance has been definitively ruled out as malignant), and long-term postoperative observation is highly advisable. Future study should prioritize molecular investigations and multicenter collaborative studies to clarify the interaction mechanisms between PNH and LCA.

## Acknowledgments

We appreciate the case for the information consent for this study.

## Author contributions

**Conceptualization**: Jun Wu.

**Formal analysis**: Sensen Zhang, Shuxian Niu.

**Investigation**: Zhonghai Du.

**Methodology: Zhenpeng** Li.

**Resources**: Zhonghai Du, Shuxian Niu.

**Software**: Zhenpeng Li.

**Supervision**: Jun Wu.

**Validation**: Sensen Zhang.
